# Supplementary material for: Patterns of Intron Gain and Loss in Fungi
Source: PLoS Biol. 2004 Nov 30;2(12):e422. doi: 10.1371/journal.pbio.0020422 (PMC532390; doi:10.1371/journal.pbio.0020422)
Supplement: Table S1 — Also available at http://genes.mit.edu/NielsenEtAl/. (4.3 MB ZIP). [file pbio.0020422.st001.zip › NielsenEtAl/html/1255.html]

AN0722.1.NCU07989.1.MG01762.1.FG05093.1


```
 CLUSTAL W (1.82) Multiple Sequence Alignments - Introns Inserted


Sequence 1: MG01762.1	143 aa
Sequence 2: FG05093.1	143 aa
Sequence 3: NCU07989.1	143 aa
Sequence 4: AN0722.1	145 aa
Alignment Length: 145 aa
Number Identitical Residues: 127 aa
Alignment Score (without introns) 5029


MG01762.1 	-MLSFILIQNRQ2GKTRLAKWYAPYS~DEEKIKLKGE0VHRLVAPRDQKYQSNFVEFR-N
NCU07989.1	-MLSFILIQNRQ2GKTRLAKWYVPYS~DEEKIKLKGE0IHRLVAPRDQKYQSNFVEFR-N
FG05093.1 	-MLSFILIQNRQ2GKTRLAKWYAPFS~DEQKIKLKGE~VHRLVAPRDQKYQSNFVEFR-N
AN0722.1  	MVLSFILVQNRQ2GKTRLAKWYAPYS0DEEKVKLKGE0VHRLVAPRDQKYQSNFVEFKRS
          	 :*****:**** *********.*:* **:*:***** :******************: .

MG01762.1 	NKLVYRRYAGLFFCACVDTNDNELAFLEAIHFFVEVLDAFFGNVCELDLVFNFYK~VYAI
NCU07989.1	HKVVYRRYAGLFFCACVDTNDNELAYLEAIHFFVEVLDSFFGNVCELDLVFNFYK~VYAI
FG05093.1 	NKIVYRRYAGLFFCACVDTNDNELAFLEAIHFFVEVLDAFFGNVCELDLVFNFYK~VYAI
AN0722.1  	TKIVYRRYAGLFFCACVDATDNELAYLEAIHFFVEVLDQFFGNVCELDLVFNFYK0VYAI
          	 *:***************:.*****:************ **************** ****

MG01762.1 	LDEVFLAGEIEETSKTVVLTRLEHLDKLE
NCU07989.1	LDEVFLAGEIEETSKQVVLTRLEHLDKLE
FG05093.1 	LDEVFLAGEIEETSKQVVLTRLEHLDKLE
AN0722.1  	LDEVFLAGEIEETSKQVVLTRLEHLDKLE
          	*************** *************
```
